# On the Recent Advances in the Theory of Vision

**Published:** 1872-02

**Authors:** H. Helmholtz

**Affiliations:** The Royal Society of London


### ART. II.—*On the Recent Advances in the Theory of Vision.* By H. HELMHOLTZ, *(of the Royal Society of London.)*

Translated from *Les Annales d'Oculistique*, BY F. W. ABBOTT, M. D.

CONTINUED.

To look at an object means, to direct the eye so that the image of the object falls upon the place of most distinct vision; it means, in other words, to make use of *direct vision,* in opposition to *indirect vision,* which is exercised by means of the lateral portions of the retina.

The lack of precision of the image, and the limited number of the sensitive elements of the retina throughout the greater part of the field of vision, are richly compensated by the eye's extreme mobility, which enables us to look successively and rapidly at all that interests us in the field of vision. Upon this great mobility, rests the principal superiority of the eye over other optical instruments. Furthermore, thanks to the peculiar manner in which our attention is exercised, it being directed to but one representation at a time, and passing constantly to new objects, the eye, as it is constructed, renders us the same services that it would if the definition of the field were the same throughout; so true is this, that those whose attention has never been called to it are usually ignorant of the imperfection of indirect vision. We look at that which interests us, and we see it clearly at each moment; the objects which we do not see in that way do not generally interest us, our attention is not directed to them, and we do not notice the imperfectness of their image.

On the contrary, it is difficult, without the long practice which certain researches in physiology require, to direct our attention to an object seen indirectly without immediately turning the eye toward this object. Our attention is, as it were, riveted to the point looked at, and the movement of the eye is inseparable from that of the attention. Again, it is no less difficult, without much practice, to fix the eye exactly upon one point for several consecutive seconds, as it is necessary to do, for example, in order to obtain sharply defined accidental images.

It is to these circumstances, doubtless, that we must attribute in great part, the importance of the role which the eye plays in the expression of the feelings. The movement of the eyes is one of the most positive signs of the movement of the attention, and consequently, also, of that of the ideas in the mind of the person observed.

The rapidity of the motions of the eye is equaled by that of the changes of the accommodation. By means of these changes the optical apparatus of the eye adapts itself for the vision of the objects at different distances, which successively attract our attention, and enables us to see them with perfect distinctness. All these changes of direction and of focus are much more rapid than they are in our unhandy optical instruments. Like the camera, the eye cannot show simultaneously with the same distinctness, objects situated at different distances; but it can pass from the distinct vision of one object to that of another with such rapidity that those who have not reflected upon the manner in which vision operates are not conscious of these changes.

Let us continue the examination of our optical apparatus. We do not wish to speak here of myopia or presbyopia, those individual anomalies of accommodation of which mention has already been made. These anomalies appear to be attributable, either to the artificial manner of living which the state of civilization induces, or to the progress of age. The faculty of accommodation passes away with the years, and distinct vision persists only for a certain determinate distance, different in different persons. To see beyond or within this distance, it is necessary to have recourse to spectacles.

There is another defect which we would not tolerate in our optical instruments. We desire that they should show no dispersion, in other words, that they should be achromatic. The colored dispersion which optical instruments present, resultsfrom the fact that the refraction of the different rays of solar light is not precisely the same, in the transparent substances which are known to us. As a consequence, the size and the situation of optical images, formed by the differently colored rays, differ a little; they cease to coincide perfectly in the field of vision of the spectator, and, according as the red rays or the blue rays give the largest image, the white surfaces appear bordered either with a yellowish red or a bluish violet, which injures, more or less, the purity of the contours.

We know the singular role which the question of the dispersion in the eye played at the time of the invention of achromatic glasses—a celebrated example of false premises leading to true conclusions. Newton believed that he had found a relation between the powers of the refraction and dispersion of different transparent substances which would render impossible the construction of achromatic instruments. Euler, admitting the achromatism of the eye, was led to contest the exactness of the relation adduced by Newton between the powers of refraction and dispersion of the different transparent substances. He then laid down theoretical rules for the construction of achromatic instruments—rules which Dolland put in practice. But Dolland at once remarked that the eye could not be achromatic, because its construction did not correspond to the rules established by Euler. Frauenhofer finally gave the numerical measurements of the amount of the dispersion. An eye, accommodated for an infinite distance in red light, is accommodated for a distance of only two feet in the violet. If we are not conscious of this dispersion in white light, it is due to the fact that the extreme colors of the spectrum are at the same time the feeblest, and that the images which they form are scarcely seen by the side of the stronger images which are formed by the yellow, red, and blue rays. On the other hand, this phenomenon becomes very remarkable when we isolate the extreme rays of the spectrum by means of violet glasses. It happens that glasses colored by oxide of cobalt transmit the red and violet rays, but extinguish the yellow and green, that is to say, the mean, and at the same time most intense colors of the spectrum. In looking from a distance at lanterns furnished with such glasses those whose vision is normal see red flames surrounded by a large blueish violet halo. This halo is the image of diffusion proceeding from the blue and violet rays of the flame. This daily phenomenon affords a convenient method of showing conclusively the existence of dispersion in the eye.

If the dispersion which takes place in the eye attracts our attention so slightly under ordinary circumstances, and if it is in reality a little less than in a glass instrument of the same power, the reason is, that in this case the principal refracting medium is water, which produces less dispersion than glass. The dispersion in the eye, however, is a little greater than would be produced by an apparatus made of pure water, other things being equal. In résumé, in spite of its existence, the dispersion does not sensibly disturb vision in ordinary white light.

*Spherical aberration* is a second defect which becomes very obvious in optical instruments of high power. Spherical refracting surfaces re-unite precisely at one point rays which proceed from one point of the object, only when these rays come almost normal to each refracting surface. To obtain an exact meeting of the rays in the central part of the image alone, it is necessary to employ surfaces differing in sphericity, and these have only recently been able to obtain with any degree of precision. Now the eye possesses surfaces which are more or less elliptical, and the prejudice in in favor of the perfection of this organ has led to attributing to them the suppression of Spherical aberration. Never did this prejudice lead to a greater error. In truth, more exact researches have discovered in the eye, aberrations much greater than that of sphericity—aberrations, which a little care enables us to avoid in artificial instruments, and by the side of which spherical aberration becomes unworthy of notice. The measurements of the curvature of the cornea, first made by Senff at Dorpat, then continued in great numbers with the aid of my ophthalmometer, by Donders, Knapp, and others, have proved that the cornea of the majority of human eyes far from having a perfectly symmetrical surface presents different curvatures in its different meridians. I have also indicated a method of examining the centration of the living eye, that is to say, of discovering whether the crystalline lens and the cornea are formed symmetrically in relation to the same axis. The application of this method has revealed in the eyes which were examined defects of centration, feeble, it is true, but incontestable. The consequences of these two kinds of aberration in the eye, known under the name of astigmatism, are found in a greater or less degree in the majority of human eyes. This defect hinders us from seeing simultaneously with perfect distinctness horizontal and vertical lines, situated at the same distance from the eye. When the astigmatism attains a considerable degree, we must have recourse to spectacle glasses of cylindrical surface, to relieve the troubles of the vision. This subject has recently attracted the attention of oculists in an especial manner.

This is not all. An elliptical refracting surface which is unsymmetrical, or a badly centred lens, shows the image of astar, at different foci, in the shape of an ellipse, a circle, or a line. In the eye, the images of luminous points are still less regular, they are surrounded by irregular rays. This phenomenon is due to the lens whose fibres are disposed in six different directions. In fact, the rays which we see around stars or distant lights are indices of the radiating structure of our lens, and the name of star, which we usually give to every radiating object, proves how wide spread is this defect. It is for the same reason that many people see the crescent of the moon double or triple when it is very narrow. If an optician should deliver to me an instrument having such imperfections, I should consider myself perfectly justified in refusing his work, and in accompanying my refusal with the harshest expressions. It is evident that I would not do so with my eyes; I desire, on the contrary, to preserve them as long as possible, in spite of their defects. If, notwithstanding their imperfections, we are glad to keep our eyes, such as they are, the optician is not less justified in criticising the imperfections they present relatively to his art.

We are far from having finished our arraignment.

If opticians wish to satisfy our wants, they must use good, perfectly transparent glass. When the glass is clouded, bright surfaces appear to be surrounded by a sort of nimbus, black appears gray, and white less clear than it ought. These defects exist in the images of the external world which the eye furnishes, and it is for this reason that we have difficulty in distinguishing dark objects situated in the neighborhood of very luminous ones. If we strongly illuminate the cornea and the crystalline of a living eye, by means of a good lamp and a convex lens, their substance appears cloudy and whitened, more cloudy than the aqueous humor which separates them. This turbidity is best seen in the blue and violet rays of the spectrum, because it is then increased by the fluorescence. The name fluorescence is given to the power which certain substances possess of manifesting a feeble glimmer of their own (*propre*) when they are illuminated by the blue or violet rays.* The bluish glimmer of solutions of quinine, and the green glimmer of uranium glass, which is yellowish green, are produced by this cause. The fluorescence of the cornea and lens appears, indeed, to be caused by a small quantity of a substance analagous to quinine which is found in their tissue. This property of the lens is, doubtless, precious to the physiologist, because by concentrating the blue rays upon it he can make the lens visible in the living eye, and prove that it is in direct contact with the posterior part of the iris, which is contrary to the long established belief on this point. But the fluorescence of the cornea and the lens can only act unfavorably upon the vision.

However clear and transparent it may appear at the moment when we have extracted it from the eye of a recently killed animal, the lens is far from being homogeneous from an optical point of

* According to Tyndall, fluorescence is the property which certain substances possess of becoming luminous when placed in the non-luminous, ultra-violet rays of the spectrum. Vide Tyndall's Notes on heat. No. 247, TRANS.

view. The shadows of the opacities and corpuscles contained in the eye, and known under the name of entoptical objects, may be made perceptible to the retina. It is sufficient for this purpose to look at a large, bright surface, as the sky, through a very small hole. The shadows of the fibres and spots of the lens appear everywhere in the images thus obtained. We see, also, all sorts of little fibres, corpuscles, and membranous folds, held in suspension in the vitreous humor, which, when they are situated near the retina, may sometimes appear to us during the ordinary use of the eye; in such cases we give them the name of muscæ volitantes because, when we wish to fix the sight upon them, these points follow the movements of the eye and consequently fly away from the point of fixation, which produces the same effect as if we saw a flying insect. There are objects of this kind in every eye; they generally float outside of the field of vision in the upper part of the eye ball, but they spread through the vitreous humor when this is agitated by any quick motion of the eye. They may sometimes settle in front of the fovea and thus obstruct the sight, It is characteristic of the way in which we observe our sensations, that persons who commence to suffer with their eyes are sometimes struck with this phenomenon which disturbs them as a new appearance, although this presence of these bodies in the vitreous was anterior to their disease.

When we know the history of the development of the eye ball in the human embryo, and in the vertebrates generally, these irregularities in the structure of the lens and the vitreous humor explain themselves. A depression is formed in the external skin of the embryo, which afterwards deepens and becomes bottle shaped; finally the neck of the bottle is entirely closed. In this little closed sac the epidermic cells unite to form the substance of the lens; the skin itself forms its capsule, and the subcutaneous connective tissue makes the vitreous humor. The cicatrix of the occlusion of the sac is often still visible entoptically in the adult.

Finally, we must not pass in silence over certain irregularities of the fundus upon which the optical image of the eye is painted. In the first place there is a hiatus in the retina, not far from the middle of the field of vision, at the spot where the optic nerve penetrates the eye ball. At this place the whole substance of the membrane is formed of fibres of the optic nerve. The cones, the elements which are sensitive to light, are entirely wanting. For this reason the light which falls upon this spot is not perceived. To this hiatus in the mosaic of the cones—called the blind spot—there corresponds a region in the field of vision in which nothing is perceived. This hiatus is far from being insignificant; it measures 6° horizontally, and 8° vertically; its internal border the one nearest the point of fixation, is situated at the temporal side of this point and about 12° distant. The easiest way to observe the blind spot is well known. Mark upon a white paper, to the left a small cross, to the right, upon the same horizontal line, and about three inches distant, a round black spot, half an inch in diameter. Close the left eye, look steadily with the right at the little cross, and slowly approach the paper, held at first at a considerable distance. At the distance of about eleven inches the black spot will disappear; it re-appears when the paper is held closer. This hiatus is large enough to contain eleven moons placed in a horizontal line, or a human face six or seven feet distant. Mariotte, who discovered this phenomenon, amused Charles II. of England, and his courtiers, by showing them how two of them could mutually see each other without a head.

A certain number of long narrow hiatuses, in which we may lose the images of very small luminous points, (such as the fixed stars,) correspond to the large vascular trunks of the retina. The vessels are situated in the anterior layers of this membrane, and consequently throw their shadows upon the parts of the sensitive mosaic which are situated behind them. The largest trunks completely prevent the light from passing through them; the smaller ones at least, weaken it. Under certain conditions these shadows of the retinal vessels may appear in the field of vision. It suffices, for example, to look at the luminous surface of the sky through a small pinhole made in a card, to which we give a slight continuous motion to and fro. It is better still by means of a small lens, to concentrate the rays of the sun upon the sclerotic near the external angle of the eye, while one looks strongly inward. The vessels of which we are speaking are situated in the anterior layers of the retina; but as their shadows can be perceived only when they touch the layer truly sensitive to light, the experiment which we have just made proves that sensitiveness to light resides in the posterior layers of the retina. This phenomenon of vascular shadows has even enabled us to measure the distance which separates the sensitive from the vascular layers of the retina. When we move the focus of the concentrated light upon the sclerotic, the shadow upon the retina also moves, and the same thing takes place with the corresponding shadow in the visual field. It is easy to measure the amount of these movements, and it is by this means that Henri Muller, of Wurtzburg, too soon taken away from science, has calculated the distance of which we have spoken above, and has found it equal to that which separates the vascular layer and that of the cones.

There is a connection in which the place of clearest vision is distinguished in a disadvantageous manner; sensitiveness to feeble light is less here than in the rest of the retina. It has been known since the most ancient times that a certain number of feebly luminous stars, such as the Hair of Berenice and the Pleiades are seen more clearly when we turn the sight a little than when we look directly at them. It can be proved that this is due, in part, to the coloring of the yellow spot, the effect of which is to weaken especially the blue light; but the want of vessels at this point, a want of which we have already spoken, may contribute to it, because from it results an obstacle to the circulation of the blood.

All these irregularities would be unendurable in an artificial camera, or in the photographs furnished by this instrument. In the eye they annoy us so little that some of them have even been difficult to discover. If they do not interfere with the perception of external objects, this is not due solely to the fact that we see with both eyes, and that the deficiencies of one eye may be supplied by the other. In fact, even for monocular vision, and in those blind of one eye, the representation of the visual field is exempt from the troubles which the irregularities of the fundus of the eye might cause. The principal reason of it is in the perpetual motion of the eye, and in the circumstance that the defects occupy almost exclusively the parts of the field of vision to which we pay no attention.

If we notice with such difficulty the phenomena of which we have just spoken, and others, such as the accidental images of bright objects, (so long as they are not sufficiently strong to interfere with the perception of surrounding objects,) it is owing to a singularity of our perception which appears paradoxical, and which exists not alone in vision, but is always found in the other senses. The history of the discovery of these phenomena is well suited to show how difficult it is to lay hold of them. Some, such as the blind spot, have been discovered by theoretical speculations. The long discussion of the question, whether sensitiveness to light resides in the retina or in the choroid, led Mariotte to ask himself how this sensitiveness behaved at the spot where the choroid was lacking. The experiments which he undertook for this purpose led him to the discovery of the hiatus in the visual field. For thousands of years men had used their eyes; many had reflected upon the purposes and the mechanism of vision, and yet a whole series of circumstances was necessary for the discovery of a phenomenon so simple that it seems as though it should have been perfectly obvious. Instead of this, every one, who for the first time makes experiments upon blind spot, experiences a certain difficulty in holding the eyes motionless while turning the attention away from the point of fixation of the sight. It is only at the price of repeated experiments that the most skillful observer becomes able, while closing one eye, to recognize immediately the place in the visual field where the hiatus is found.

Other phenomena of the same kind have been discovered by chance, and this especially by men particularly endowed by nature with the faculty of observation which these researches demand. We name in the first rank, Goethe, Purkinje and Jean Muller. To recognize in one's own eyes a phenomenon already known is much easier than to discover it anew; nevertheless a great part of the phenomena which Purkinje describes have not been seen again by others, yet we cannot say with certainty that they were peculiar to the eyes of this eminent observer.

The phenomena of which we have just spoken, and many others, are governed by this general rule, that a change in the degree of irritation of a sensitive nerve is perceived much more easily than a constant and invariable irritation. As a consequence of this rule, the peculiarities in the irritation of certain fibres which are the same throughout life—as, for example, the vascular shadows in the eye, the yellow color of the centre of the retina, and the majority of the motionless entoptical objects—entirely escape our attention, and in order to make them appear, it is necessary to have recourse to an unusual illumination, whose direction, moreover, it is useful to continually vary.

According to what we know at present of irritation of nerves, it appears to me scarcely probable that we have to do here with a phenomenon of sensation merely; I believe, rather, that it is a phenomenon of observation. It suffices to have noticed this here, for the solution of the questions which we meet on our way will find its place farther on.

We will not dwell longer on the physical functions of the eye. If I should be asked why I have said so much of its imperfections, I would answer that this has not been to depreciate this little organ and defraud it of the admiration which it deserves. My chief desire was simply to demonstrate first and foremost, that it is not to the mechanical perfection of the organs of the senses that the fidelity and marvelous accuracy of the impressions which they furnish must be attributed. The next part of this work will show still bolder and more paradoxical incongruities. We have now seen that the eye is not of itself so perfect an optical instrument as it appears, but that it renders us such good service only from the special manner in which we use it. Its perfection is purely practical, and by no means absolute; it does not consist in the fact that every defect is avoided, but that the defects do not hinder its most useful and varied applications.

In this relation the study of the eye enables us to investigate deeply the character of organic perfection in general, and the interest of these researches increases when they are placed in connection with the vast and bold ideas which Darwin has just introduced into science, relatively to the character of the progressive development of the organs. Wherever we study organic formations, we encounter the same character of adaptation to the end to be attained, but it is perhaps in the eye that we can better than any where else, assure ourselves in detail that this is so. The eye contains all the defects which optical instruments present, and even some which we not tolerate in these instruments; but these defects are all kept would within such limits, that the inaccuracy which they cause in the image scarcely surpasses in the ordinary condition of illumination, the limit imposed on the acuteness of the perception by the delicacy of the cones, the elements sensitive to light. For all that we have observed to the contrary, under slightly different conditions we would notice the dispersion, the astigmatism, the hiatus, the vascular shadows, the imperfect transparency of the media, etc.

So we see the perfect adaptation of the eye to its purpose, and this is revealed even in the limits which are set to its defects. That which the labor of an innumerable succession of generations has been able to produce under the influence of Darwin's law of descent is doubtless here joined to a work created by infinite Wisdom. A reasonable man will not take a razor to chop wood; so we may allow that each useless refinement in the optical construction of the eye would have rendered this organ more delicate, and slower in its development. Neither should we forget that the animal tissues, soft and full of water, are unpromising materials for the construction of an instrument of physics.

This state of things is the reason why perception only occurs clearly and without obstacle in turning the eye about the field of vision in the way to which we have alluded, a circumstance whose importance will be shown subsequently. We will also speak further on of other circumstance which act in the same direction.

Up to this point we do not appear to have made much progress in the question of how vision takes place. All that we have learned is how the optical apparatus unravels the light which comes to it from the different points of the field of vision, and distributes it so as to deliver to one single sensitive fibre all that proceeds from one single external point.

Let us see now if what we know of the sensations of the eye will bring us nearer to the solution of this problem.

[TO BE CONTINUED.]

